# Glutamatergic transmission in drug reward: implications for drug addiction

**DOI:** 10.3389/fnins.2015.00404

**Published:** 2015-11-05

**Authors:** Manoranjan S. D'Souza

**Affiliations:** Pharmaceutical and Biomedical Sciences, Raabe College of Pharmacy, Ohio Northern UniversityAda, OH, USA

**Keywords:** cocaine, nicotine, alcohol, heroin, reward, nucleus accumbens, prefrontal cortex, microdialysis

## Abstract

Individuals addicted to drugs of abuse such as alcohol, nicotine, cocaine, and heroin are a significant burden on healthcare systems all over the world. The positive reinforcing (rewarding) effects of the above mentioned drugs play a major role in the initiation and maintenance of the drug-taking habit. Thus, understanding the neurochemical mechanisms underlying the reinforcing effects of drugs of abuse is critical to reducing the burden of drug addiction in society. Over the last two decades, there has been an increasing focus on the role of the excitatory neurotransmitter glutamate in drug addiction. In this review, pharmacological and genetic evidence supporting the role of glutamate in mediating the rewarding effects of the above described drugs of abuse will be discussed. Further, the review will discuss the role of glutamate transmission in two complex heterogeneous brain regions, namely the nucleus accumbens (NAcc) and the ventral tegmental area (VTA), which mediate the rewarding effects of drugs of abuse. In addition, several medications approved by the Food and Drug Administration that act by blocking glutamate transmission will be discussed in the context of drug reward. Finally, this review will discuss future studies needed to address currently unanswered gaps in knowledge, which will further elucidate the role of glutamate in the rewarding effects of drugs of abuse.

## Introduction

Rewards increase motivation to perform or repeat tasks and can be broadly classified as natural and drug rewards (Schultz, 2006). Natural rewards are critical for survival and include food, water, and sex. In contrast, drug rewards are consumed for their ability to produce pleasure and euphoria. Although both natural and drug rewards activate similar systems in the brain, the stimulation of reward systems by drug rewards is often much more powerful than that produced by natural rewards (Wise, 1987; Koob, 1992a; Berridge and Robinson, 1998; Kelley and Berridge, 2002; Dileone et al., 2012). Furthermore, changes in neuronal communication induced by drug rewards in the brain are so powerful that they can alter controlled social use of a substance into uncontrolled compulsive use in vulnerable individuals (Koob et al., 2004 but also see Pelchat, 2009; Volkow et al., 2013). This transition to uncontrolled compulsive use is termed addiction, which results in significant mortality and morbidity all over the world.

Drug rewards can be broadly classified into licit (e.g., alcohol and nicotine) and illicit (e.g., cocaine, heroin) substances. These drugs can also be classified based on their effects in humans as stimulants (cocaine and nicotine) and depressants (alcohol and heroin). Irrespective of the type of drug, the rewarding effects associated with drugs of abuse play a role in the initiation and maintenance of the drug taking habit (Wise, 1987). Therefore, identifying neural substrates that mediate the rewarding effects of drugs of abuse will help in our understanding of processes involved in the development of drug addiction and help in the discovery of medications for its treatment.

Over the last three decades, the role of the excitatory neurotransmitter glutamate has been extensively studied in several aspects of drug addiction, including drug reward. Interestingly, some recent studies have demonstrated that glutamate may be involved in mediating natural reward as well (Bisaga et al., 2008; Pitchers et al., 2012; Mietlicki-Baase et al., 2013). However, this review will restrict its focus on the role of glutamate in drug reward. Specifically, the review will describe the role of glutamate in the rewarding effects of drugs such as cocaine, nicotine, alcohol, and heroin. First the effects of glutamate transmission blockade on behavioral measures of drug reward will be discussed. Next, the role of glutamate in specific brain sites such as the ventral tegmental area (VTA) and nucleus accumbens (NAcc), which are associated with the rewarding effects of drugs of abuse will be discussed. Finally, the review will discuss gaps in knowledge that may be addressed by future studies with respect to the role of glutamate in drug reward.

## Behavioral measures of the rewarding/reinforcing effects of drugs of abuse

In this review, discussion will be restricted to three models commonly used to assess the rewarding effects of drugs of abuse. These include drug self-administration, drug-induced conditioned place preference (CPP), and intracranial self-stimulation (ICSS). Drug self-administration is the most robust and reliable model to measure the rewarding effects of drugs of abuse (O'Connor et al., 2011). Drug self-administration can be operant (e.g., animal has to press a lever or poke its nose into a designated hole) or non-operant (e.g., oral consumption of a drug when presented with a choice of drug and non-drug bottles). Operant drug self-administration is commonly used to assess the reinforcing effects of nicotine, cocaine, alcohol, and heroin, while non-operant self-administration is used to assess the reinforcing effects of alcohol. Operant drug self-administration involves either fixed- or progressive-ratio schedules. Fixed-ratio schedules, in which the animal has to press a lever (or poke its nose into a particular hole) a fixed number of times to obtain the drug, are generally used to measure the reinforcing effects of a drug. In contrast, progressive-ratio schedules, which require increasing responses to obtain each successive drug infusions/delivery, are used for measuring the motivational effects of a drug. The main measure determined by the progressive-ratio schedules is the break point, defined as the number of ratios completed by the subject per session. In other words, break point, reflects the maximum work an animal will perform to obtain another infusion/delivery of the drug. Several studies have demonstrated reliable intravenous self-administration of cocaine, nicotine, and heroin under both fixed- and progressive-ratio schedules (e.g., Roberts and Bennett, 1993; Duvauchelle et al., 1998; Paterson and Markou, 2005). Additionally, several studies have demonstrated oral self-administration of alcohol using the two bottle choice paradigm (e.g., Grant and Samson, 1985; Pfeffer and Samson, 1985; Samson and Doyle, 1985; Suzuki et al., 1988).

The rewarding effects of drugs of abuse can also be studied using the CPP procedure (for review see Tzschentke, 2007). In this procedure, the preference of an animal for a drug-paired environment is compared to its preference for a vehicle (control)-paired environment. Typically, the apparatus used for the procedure consists of atleast two chambers with distinct characteristics (e.g., color, texture, flooring). The animal is initially given a choice to explore both chambers, and the time spent by the animal in each chamber is noted. Subsequently, during training, the animal is consistently confined to one of the two chambers (drug-paired chamber) after administration of drug of abuse being studied. In another temporally distinct training session, the animal is treated with vehicle (control) and placed in the other chamber, referred to as the vehicle-paired chamber. After several pairings of the drug and vehicle with the drug- and vehicle-paired chamber, respectively, the animal is given a chance to simultaneously explore both chambers during a test session. The repeated pairing of the drug-paired chamber with the rewarding effects of the drug over time results in a preference for the drug-paired chamber compared to the vehicle-paired chamber during the test session, reflected by the animal spending more time in the drug-paired chamber. Notably, the test session is conducted without administration of the drug of abuse being studied. Several studies have demonstrated CPP with cocaine, nicotine, alcohol, and heroin (e.g., Reid et al., 1985; Schenk et al., 1985; Nomikos and Spyraki, 1988; Le Foll and Goldberg, 2005; Xu et al., 2015).

The rewarding effects of drugs of abuse can also be assessed using ICSS, which involves stimulation of brain reward circuits using brief electrical pulses (Markou and Koob, 1993). In this procedure, animals are surgically implanted with electrodes, which stimulate discrete brain areas associated with reward (e.g., the lateral hypothalamus or NAcc). After recovery from surgery, the animals are trained to self-stimulate using brief electrical currents of different strengths. Once the animals are trained, a reward threshold, defined as the minimal strength of the electric current required to maintain self-stimulation behavior, is determined. Administration of drugs of abuse lower the reward threshold required to maintain ICSS behavior (e.g., Kornetsky and Esposito, 1979; Harrison et al., 2002; Gill et al., 2004; Kenny et al., 2009).

In summary, several different animal models are available to assess the rewarding effects of drugs of abuse. The readers are referred to other scholarly work for detailed discussion of these and other models to assess the rewarding effects of drugs of abuse (for review see Brady, 1991; Markou and Koob, 1993; Sanchis-Segura and Spanagel, 2006; Tzschentke, 2007; Negus and Miller, 2014). The subsequent sections of the review will focus on the role of glutamate in drug reward, which has been elucidated using the above described animal models.

## Glutamate and drugs of abuse

### General overview of glutamate transmission

Glutamate is the major excitatory neurotransmitter in the mammalian brain and accounts for approximately 70% of synaptic transmission in the central nervous system (Nicholls, 1993; Niciu et al., 2012). The actions of glutamate are mediated by both fast-acting ligand-gated ion channels, commonly referred to as ionotropic glutamate receptors, and slow-acting G-protein coupled receptors also known as metabotropic glutamate (mGlu) receptors (Wisden and Seeburg, 1993; Niswender and Conn, 2010). The ionotropic glutamate receptors include N-methyl-D-aspartate (NMDA), amino-3-hydroxy-5-methyl-4-isoxazolepropionate (AMPA), and kainate receptors. NMDA receptors are heterotetramers composed of NR1, NR2 (NR2A-D), and rarely NR3 subunits (Zhu and Paoletti, 2015). NMDA receptors are complex receptors and require binding of glutamate, the co-agonist glycine, and membrane depolarization for removal of a magnesium block. This membrane depolarization occurs via activation of AMPA receptors, which are described as workhorses amongst the glutamate receptors. AMPA receptors are also tetramers and are composed of GluR 1–4 subunits (Hollmann and Heinemann, 1994). Unique subunit combinations confer differential glutamate signaling properties to the NMDA and AMPA receptors.

In addition to the ionotropic receptors, eight mGlu receptors have been identified and are classified into three Groups (I, II, and III) depending on their signal transduction pathways, sequence homology, and pharmacological selectivity (Pin and Duvoisin, 1995; Niswender and Conn, 2010). Group I (mGlu1 and mGlu5) receptors are predominantly located postsynaptically, and Group II (mGlu2 and mGlu3) and Group III (mGlu4, mGlu6, mGlu7, and mGlu8) receptors are primarily found on presynaptic glutamate terminals and glial cells. Notably, the Group II and III mGlu receptors negatively regulate glutamate transmission, i.e., activation of these receptors decreases glutamate release. In other words, an agonist or positive allosteric modulator at Group II or III mGlu receptors decreases glutamate transmission. There is an increasing focus on the role of metabotropic receptors in drug reward and addiction (Duncan and Lawrence, 2012). Activation of either the ionotropic or mGlu receptors results in stimulation of a number of intracellular signaling pathways, ultimately leading to neuronal plasticity. In fact, drug-induced plasticity in glutamatergic transmission is critically involved in the development of drug addiction (Kalivas, 2004, 2009; van Huijstee and Mansvelder, 2014).

Extracellular glutamate is cleared from the synapse by excitatory amino acid transporters (EAATs) and vesicular glutamate transporters (VGLUTs). The EAATs are located on glutamate terminals and presynaptic glial cells and play an important role in glutamate homeostasis (O'Shea, 2002; Kalivas, 2009). Till date several different types of EAATs have been reported in animals (GLT-1, GLAST, and EAAC1) and humans (EAAT1, EAAT2, and EAAT3) (Arriza et al., 1994). VGLUTs are mainly responsible for the uptake and sequestration of glutamate into presynaptic vesicles for storage. So far three different isoforms of VGLUTs (VGLUT1, VGLUT2, and VGLUT3) have been discovered (El Mestikawy et al., 2011). Glutamate can also be transported back into the extrasynaptic space via the cystine-glutamate antiporter located on glial cells (Lewerenz et al., 2012). The cystine-glutamate antiporter exchanges extracellular cystine for intracellular glutamate and serves as a source non-vesicular glutamate release. Glutamate transporters can serve as targets for attenuating the rewarding effects of drugs of abuse (Ramirez-Niño et al., 2013; Rao et al., 2015a).

### Drugs of abuse and alteration of glutamate transmission

Drugs of abuse alter glutamate transmission via different mechanisms. The primary site of action of cocaine is the dopamine uptake transporter (DAT; Ritz et al., 1987). Cocaine blocks DAT and increases dopamine levels, which mediates the rewarding effects of cocaine. Cocaine-induced increase in synaptic dopamine levels activates presynaptic or postsynaptic D1 dopamine receptors, which indirectly increases glutamate transmission. Activation of presynaptic D1 receptors regulates cocaine-induced increase in glutamate levels (Pierce et al., 1996b). Additionally, dopamine can bind to postsynaptic D1 receptors and regulates ionotropic glutamate transmission via the NMDA and AMPA receptors (for review see Wolf et al., 2003). For example, D1 receptor activation increases AMPA receptor trafficking and insertion into the membrane via protein kinase A-mediated phosphorylation (Gao and Wolf, 2007). Further, activation of D1 receptors increases NMDA-mediated glutamate signaling via either increased insertion in the postsynaptic membrane or functional cross-talk between D1 and NMDA receptors (Dunah and Standaert, 2001; Ladepeche et al., 2013).

On the other hand nicotine, another stimulant, increases glutamate transmission by binding to excitatory α7 homomeric nicotinic acetylcholine receptors located on presynaptic glutamate terminals (Mansvelder and McGehee, 2000). In addition, nicotine possibly increases glutamate signaling via dopaminergic mechanisms such as those described for cocaine (Mansvelder et al., 2002). In summary, psychostimulants like cocaine and nicotine increase glutamate transmission without directly interacting with glutamate receptors.

Studies using patch-clamp and other electrophysiological techniques in brain slices report that alcohol inhibits postsynaptic NMDA- and non-NMDA-mediated glutamate transmission (Lovinger et al., 1989, 1990; Nie et al., 1993; Carta et al., 2003). Further, electrophysiology studies suggest that alcohol inhibits presynaptic glutamate release (Hendricson et al., 2003, 2004; Ziskind-Conhaim et al., 2003). Conversely, using *in vivo* microdialysis, some studies report an increase in glutamate levels after alcohol administration (Moghaddam and Bolinao, 1994). This alcohol-induced increase in glutamate release is possibly due to inhibition of GABAergic interneurons that in turn inhibit presynaptic glutamate terminals. Another presynaptic mechanism for alcohol-induced increase in glutamate transmission could be via activation of D1 receptors (Deng et al., 2009; for review see Roberto et al., 2006). Electrophysiological studies suggest that repeated exposure to alcohol facilitates presynaptic and postsynaptic glutamate transmission (Zhu et al., 2007).

Finally, heroin, which mainly binds to mu opioid receptors, alters glutamate transmission via several different mechanisms. For example, activation of mu opioid receptors decreases NMDA- and non-NMDA mediated glutamate transmission via presynaptic mechanisms (Martin et al., 1997). Further, direct interaction between mu opioid receptors and NMDA receptors has been demonstrated in several brain regions (Rodriguez-Muñoz et al., 2012). Interestingly, mu-opioid receptor activation increases postsynaptic NMDA-mediated glutamate transmission via activation of protein kinase C (Chen and Huang, 1991; Martin et al., 1997). Heroin, similar to alcohol, can potentially increase glutamate transmission by inhibiting GABAergic interneurons, which inhibit presynaptic glutamate terminals (Xie and Lewis, 1991). Finally, heroin can increase glutamate signaling indirectly via dopaminergic mechanisms as described above for cocaine (for review see Svenningsson et al., 2005; Chartoff and Connery, 2014).

In summary, amongst the drugs of abuse being discussed in this review, only alcohol directly interacts with the glutamate receptors. The other drugs of abuse discussed in this review alter glutamate transmission indirectly via presynaptic and postsynaptic mechanisms. In the subsequent section, we will discuss the effects of blocking glutamatergic transmission using pharmacological compounds on behavioral measures of drug reward.

### Blockade of glutamatergic transmission and behavioral measures of drug reward

Systemic administration of pharmacological compounds that block glutamate transmission attenuated the reinforcing effects of drugs of abuse (see Table 1). For example, systemic administration of NMDA receptor antagonists attenuated self-administration of cocaine (Pierce et al., 1997; Pulvirenti et al., 1997; Hyytiä et al., 1999; Allen et al., 2005; Blokhina et al., 2005; but see also Hyytiä et al., 1999), alcohol (Shelton and Balster, 1997), and nicotine (Kenny et al., 2009). Additionally, systemic administration of the NMDA receptor antagonists attenuated cocaine- and alcohol-induced CPP (Cervo and Samanin, 1995; Biala and Kotlinska, 1999; Boyce-Rustay and Cunningham, 2004; Maldonado et al., 2007) as well as nicotine-induced lowering of ICSS thresholds (Kenny et al., 2009). Together, the above studies support a role for NMDA receptors in the rewarding effects of cocaine, nicotine and alcohol. Interestingly, systemic administration of NMDA receptor antagonists increased heroin self-administration. The increase in heroin self-administration was however observed in the first hour of a three hour self-administration session, thus suggesting that the increase in heroin self-administration may be an attempt to compensate for the decrease in the rewarding effects of heroin (Xi and Stein, 2002). Alternatively, NMDA-mediated glutamate transmission may have a differential role in the reinforcing effects of heroin compared to cocaine, nicotine, and alcohol. Further work using a progressive-ratio schedule will be required, to determine if NMDA receptor blockade increases or decreases the rewarding effects of heroin. In summary, one can conclude that systemic administration of NMDA receptor antagonists generally attenuates the rewarding effects of drugs of abuse.

**Table 1:** **Effects of pharmacological manipulation of glutamatergic transmission on behavioral measures of drug reward**.

**Drug of abuse**	**Glutamate receptor/Transporter**	**Pharmacological class**	**Ligand**	**Species/Strain**	**Model used to assess reward**	**Effect observed**	**References**
Cocaine	NMDA	Antagonist	Dizocilpine (MK-801)	Rats/Sprague-Dawley	Self-administration	Decreased	Pierce et al., 1997
			Dextromethorphan	Rats/Wistar	Self-administration	Decreased	Pulvirenti et al., 1997
			Dizocilpine (MK-801)	Rats/Wistar	Self-administration	Decreased	Hyytiä et al., 1999
			Memantine	Rats/Wistar	Self-administration	Decreased	Hyytiä et al., 1999
			CGP39551	Rats/Wistar	Self-administration	No effect	Hyytiä et al., 1999
			L-701,324	Rats/Wistar	Self-administration	No effect	Hyytiä et al., 1999
			LY235959	Rats/Sprague-Dawley	Self-administration	Decreased	Allen et al., 2005
			Memantine	Mice/Swiss	Self-administration	Decreased	Blokhina et al., 2005
			Dizocilpine (MK-801)	Rats/Sprague-Dawley	CPP	Decreased	Cervo and Samanin, 1995
			Memantine	Mice/Albino OF1 strain	CPP	Decreased	Maldonado et al., 2007
		Partial agonist at glycine receptor site	(+)-HA-966	Rats/ Sprague-Dawley	Self-administration	Decreased	Cervo et al., 2004
			ACPC	Rats/Wistar	CPP	Decreased	Papp et al., 2002
			D-cycloserine	Mice/C57/BL6	CPP	Decreased	Yang et al., 2013
	AMPA	Antagonist	DNQX	Rats/Sprague-Dawley	Self-administration	Decreased	Pierce et al., 1997
	mGlu2/3	Agonist	LY379268	Rats/Wistar	Self-administration	Decreased	Baptista et al., 2004
				Monkeys/Squirrel monkeys	Self-administration	Decreased	Adewale et al., 2006
		Endogenous agonist	N-acetylaspartylglutamate	Rats/Long Evans	Self-administration	Decreased	Xi et al., 2010
	mGlu2	Positive allosteric modulator	BINA	Rats/Wistar	Self-administration	Decreased	Jin et al., 2010
		Positive allosteric modulator	SB-44	Rats/Wistar	Self-administration	Decreased	Dhanya et al., 2014
	mGlu5	Negative allosteric modulator	MPEP	Rats/Wistar	Self-administration	Decreased	Tessari et al., 2004
			MPEP	Rats/Wistar	Self-administration	Decreased	Kenny et al., 2005
			MTEP	Rats/Wistar	Self-administration	Decreased	Martin-Fardon et al., 2009
			MPEP	Rats/Sprague-Dawley	CPP	No effect	Herzig and Schmidt, 2004
			MTEP	Rats/Wistar	CPP	No effect	Veeneman et al., 2011
			MPEP	Rats/Sprague-Dawley	CPP	Increased	Rutten et al., 2011
	mGlu7	Positive allosteric modulator	AMN082	Rats/Long Evans	Self-administration	Decreased	Li et al., 2009
	GLT-1	Activator	MS-153	Mice/ddY	CPP	Decreased	Nakagawa et al., 2005
Nicotine	NMDA	Antagonist	LY235959	Rats/Wistar	Self-administration	Decreased	Kenny et al., 2009
		Partial agonist	D-cycloserine	Rats/Sprague- Dawley (female)	Self-administration	Decreased	Levin et al., 2011
		Partial agonist	ACPC	Rats/Wistar	CPP	Decreased	Papp et al., 2002
	mGlu2/3	Agonist	LY379268	Rats/Wistar	Self-administration	Decreased	Liechti et al., 2007
	mGlu2	Positive allosteric modulator	BINA	Rats/Wistar	Self-administration	Decreased	Sidique et al., 2012
	mGlu5	Negative allosteric modulator	MPEP	Rats/Wistar	Self-administration	Decreased	Paterson et al., 2003
			MPEP	Rats/Wistar	Self-administration	Decreased	Tessari et al., 2004
			MPEP and MTEP	Rats/Sprague-Dawley	Self-administration	Decreased	Palmatier et al., 2008
			MPEP	Rats/Sprague-Dawley	CPP	Increased	Rutten et al., 2011
	Cystine-Glutamate transporter	Activator	N-acetylcysteine	Rats/Wistar	Self-administration	Decreased	Ramirez-Niño et al., 2013
Alcohol	NMDA	Antagonist	CPP	Rats/LongEvans	Self-administration	Decreased	Shelton and Balster, 1997
			Dizocilpine (MK-801)	Rats/Wistar	CPP	Decreased	Biala and Kotlinska, 1999
			L-701,324	Rats/Wistar	CPP		
			CGP-37849	Mice/DBA/2J	CPP	Decreased	Boyce-Rustay and Cunningham, 2004
			Dizocilpine (MK-801)	Mice/DBA/2J	CPP	No effect	Boyce-Rustay and Cunningham, 2004
			Ketamine	Mice/DBA/2J	CPP	No effect	Boyce-Rustay and Cunningham, 2004
			Ifenprodil (NR-2 B selective)	Mice/DBA/2J	CPP	No effect	Boyce-Rustay and Cunningham, 2004
			CP-101,606	Mice/DBA/2J	CPP	No effect	Boyce-Rustay and Cunningham, 2004
		Partial agonist at glycine receptor site	(+)-HA-966	Mice/DBA/2J	CPP	No effect	Boyce-Rustay and Cunningham, 2004
	AMPA	Antagonist	NBQX	Rats/Lister hooded	Self-administration	Decreased	Stephens and Brown, 1999
			GYKI 52466			No effect	
	mGlu1	Antagonist	EMQMCM	Rats/Wistar	CPP	Decreased	Kotlinska et al., 2011
	mGlu2/3	Agonist	LY379268	Rats/Long-Evans	Self-administration	Decreased	Bäckström and Hyytia, 2005
			LY379268	Rats/Wistar			Sidhpura et al., 2010
	mGlu2	Antagonist	LY341495	Rats/Wistar	Oral Self-administration	Increased	Zhou et al., 2013
	mGlu5	Negative allosteric modulator	MPEP	Rats/Alcohol preferring (P)	Self-administration	Decreased	Schroeder et al., 2005
			MPEP	Mice/C57BL/6J	Self-administration	Decreased	Olive et al., 2005
			MPEP		Self-administration	Decreased	Hodge et al., 2006
			MPEP	Mice/WSC	Self-administration	Decreased	Tanchuck et al., 2011
			MTEP	Rats/Wistar	Self-administration	Decreased	Sidhpura et al., 2010
			MTEP	Rats/Wistar	CPP	Decreased	Kotlinska et al., 2011
	mGlu7	Positive allosteric modulator	AMN-082	Rats/Wistar	CPP	Decreased	Bahi et al., 2012
	mGlu8	Agonist	(S)-3,4-DCPG	Rats/Long-Evans	Self-administration	Decreased	Bäckström and Hyytia, 2005
	GLT-1	Compounds produce upregulation of GLT-1	Ceftriaxone	Rats/Alcohol preferring (P)	Oral self-administration using a two bottle choice paradigm	Decreased	Sari et al., 2011
			Ampicillin	Rats/Alcohol preferring (P)	Oral self-administration using a two bottle choice paradigm	Decreased	Rao et al., 2015b
			Cefazolin	Rats/Alcohol preferring (P)	Oral self-administration using a two bottle choice paradigm	Decreased	Rao et al., 2015b
			Cefoperazone	Rats/Alcohol preferring (P)	Oral self-administration using a two bottle choice paradigm	Decreased	Rao et al., 2015b
			MS-153	Rats/Alcohol preferring (P)	Oral self-administration using a two bottle choice paradigm	Decreased	Alhaddad et al., 2014b
			GPI-1046	Rats/Alcohol preferring (P)	Oral self-administration using a two bottle choice paradigm	Decreased	Sari and Sreemantula, 2012
Heroin	NMDA	Antagonist	Dizocilpine (MK-801)	Rats/Sprague-Dawley	Self-administration	Increased	Xi and Stein, 2002
			Ketamine	Rats/Sprague-Dawley	Self-administration	Increased	Xi and Stein, 2002
	AMPA	Positive allosteric modulator	Piracetam	Rats/Sprague-Dawley	CPP	Increased	Xu et al., 2015
	mGlu5	Negative allosteric modulator	MPEP	Rats/Long-Evans	Self-administration	Decreased	van der Kam et al., 2007
				Rats/Sprague Dawley	CPP	Increased	van der Kam et al., 2009a

Intriguingly, several animal studies have shown that NMDA receptors have rewarding effects of their own (Carlezon and Wise, 1993). Further in humans, NMDA receptor antagonists induce a psychosis-like state (Malhotra et al., 1996). The psychotic effects, though, are less pronounced or even absent in some NMDA receptor antagonists and NMDA receptor antagonists have been approved for use in humans. For example, the FDA has approved memantine, a non-competitive NMDA antagonist, for the treatment of Alzheimer's disease (Cummings, 2004). Interestingly, clinical studies report that memantine decreased the positive subjective effects of cigarette smoking and intravenous heroin in human subjects (Comer and Sullivan, 2007; Jackson et al., 2009). In contrast, high doses of memantine increased the subjective effects of cocaine in humans (Collins et al., 1998). Acamprosate, a FDA approved medication for the treatment of alcohol use disorder, decreases glutamatergic transmission by blocking NMDA-mediated glutamate transmission (Rammes et al., 2001; Mann et al., 2008; but see Popp and Lovinger, 2000). In animals, acamprosate attenuated the rewarding effects of alcohol and cocaine (Olive et al., 2002; McGeehan and Olive, 2003a). Finally, another non-competitive NMDA antagonist called ketamine, not yet approved by the FDA, has shown promise in the treatment of severely depressed patients (for review see Coyle and Laws, 2015). Together, the above described medications suggest that NMDA receptor is a viable target for future drug development.

NMDA-mediated glutamate transmission can be disrupted using other approaches. One such approach may be the use of subunit selective NMDA receptor antagonists such as ifenprodil, which is selective for the NR2B subunit of the NMDA receptor. Administration of ifenprodil did not reduce oral alcohol self-administration or alcohol-induced CPP (Yaka et al., 2003). However, the role of specific NMDA receptor subunits in rewarding effects of other drugs of abuse has not been systematically addressed. Currently, the lack of NMDA subunit-specific pharmacological ligands is an impediment to the systemic assessment of the role of NMDA receptors composed of different subunits in drug reward. NMDA-mediated glutamate transmission can also be decreased by manipulating the glycine site of the NMDA receptors. Glycine is a co-agonist required for activation of the NMDA receptor and administration of a partial agonist that binds to the glycine site of the NMDA receptor decreased self-administration of cocaine (Cervo et al., 2004) and nicotine (Levin et al., 2011). Further, ACPC, a partial agonist at the glycine site of the NMDA receptor, attenuated cocaine- and nicotine-induced CPP (Papp et al., 2002; Yang et al., 2013).

Decrease in ionotropic-mediated glutamate transmission via blockade of AMPA receptors attenuated self-administration of cocaine (Pierce et al., 1997) and alcohol (Stephens and Brown, 1999). In addition, activation of AMPA receptors facilitated heroin-induced CPP (Xu et al., 2015). Together, these studies support a role for the AMPA receptors in drug reward. Topiramate, a FDA-approved anti-epileptic medication, attenuates AMPA-mediated glutamate transmission (Gryder and Rogawski, 2003). Relevant to this review, administration of topiramate decreased consumption of alcohol in C57BL/6J mice compared to vehicle, further supporting a role for the AMPA receptors in the reinforcing effects of alcohol. Notably, in abstinent human smokers, topiramate treatment increased the subjective effects of cigarette smoking. This enhancement in rewarding effects of cigarette smoking could be due to an increase in nicotine withdrawal effects in abstinent smokers (Reid et al., 2007). In support of this hypothesis, a study reported that blockade of AMPA receptors induced aversive withdrawal-like effects in nicotine-dependent rats (Kenny et al., 2003). More recently, a preliminary study reported that topiramate compared to placebo resulted in higher quit rates amongst smokers (Oncken et al., 2014). In addition to blocking AMPA receptors, topiramate can act via other mechanisms including blockade of presynaptic voltage-gated calcium and sodium ion channels, which must be kept in mind while interpreting the findings of the above described studies (Rosenfeld, 1997). Considering that drugs of abuse, especially psychostimulants, significantly affect AMPA-receptor trafficking (Wolf, 2010), it is surprising that the role of AMPA receptors in drug reward has not been extensively studied. Future studies targeting specific AMPA receptor subunits may help in better understanding of the role of AMPA receptors in drug reward. More recently, the FDA approved a non-competitive AMPA receptor antagonist, perampanel, for the treatment of epilepsy. Although the effects of perampanel on drug reward have not been explored, the approval of an AMPA receptor antagonist for clinical use suggests that the AMPA receptors may be a safe and viable target for the discovery and development of drugs targeting drug reward and the treatment of drug addiction.

Blockade of glutamate transmission via mGlu receptors also attenuated the rewarding effects of drugs of abuse. Blockade of mGlu1 receptors attenuated alcohol-induced CPP (Kotlinska et al., 2011). The role of mGlu1 receptors in the rewarding effects of other drugs of abuse has not been explored. Blocking glutamate transmission via the mGlu5 receptor using mGlu5 receptor negative allosteric modulators MPEP or MTEP attenuated self-administration of cocaine (Tessari et al., 2004; Kenny et al., 2005; Martin-Fardon et al., 2009; Keck et al., 2014), nicotine (Paterson et al., 2003; Paterson and Markou, 2005; Liechti and Markou, 2007; Palmatier et al., 2008), alcohol (Olive et al., 2005; Schroeder et al., 2005; Hodge et al., 2006; Tanchuck et al., 2011), and heroin (van der Kam et al., 2007). Further, the blockade of mGlu5 receptors using the above compounds attenuated cocaine- and nicotine-induced CPP (McGeehan and Olive, 2003b; Herzig and Schmidt, 2004; Yararbas et al., 2010). In summary, the above studies suggest that mGlu5-mediated glutamate transmission mediates the rewarding effects of cocaine, nicotine, alcohol, and heroin.

On the other hand, not all studies are consistent with respect to the role of mGlu5 receptors in drug reward. For example, blockade of mGlu5 receptors using the negative allosteric modulators MPEP or MTEP had no effects on nicotine- and cocaine-induced CPP, respectively (Herzig and Schmidt, 2004; Veeneman et al., 2011). In contrast, another study found that mGlu5 negative allosteric modulator MPEP facilitated cocaine-, nicotine-, and heroin-induced CPP (van der Kam et al., 2009a; Rutten et al., 2011). Furthermore, MPEP was self-administered by rats and induced CPP when administered alone in rats (van der Kam et al., 2009b). These findings indicate that MPEP probably has rewarding properties of its own, which possibly facilitated cocaine-, nicotine-, and heroin-induced CPP. Surprisingly, when administered intraperitoneally, MPEP elevated brain reward thresholds, suggesting that MPEP induced an aversive state (Kenny et al., 2005). These conflicting findings could be due to methodological differences between the studies, such as strains of animals used, doses of MPEP, mode of administration (intravenous vs. intraperitoneal), model used to assess reward (CPP vs. ICSS), and design of the CPP model itself. Finally, MPEP can act via other targets such as norepinephrine transporters and mGlu4 receptors (Heidbreder et al., 2003; Mathiesen et al., 2003). Further work is required to understand the role of mGlu5 receptors in the rewarding effects of drugs of abuse.

As described earlier, activation of Group II (mGlu2/3) and Group III (mGlu7 and mGlu8) mGlu receptors decreases glutamate transmission. In accordance, administration of the mGlu2/3 agonist LY379268 decreased self-administration of cocaine (Baptista et al., 2004; Adewale et al., 2006; Xi et al., 2010), nicotine (Liechti et al., 2007), and alcohol (Bäckström and Hyytia, 2005; Sidhpura et al., 2010). Further elevation of N-acetylaspartylglutamate (NAAG), an endogenous agonist of the mGlu2/3 receptors, using a NAAG peptidase inhibitor, attenuated cocaine self-administration and cocaine-induced lowering of brain reward thresholds (Xi et al., 2010). Together, these studies point to an important role for mGlu2/3 receptors in the reinforcing effects of cocaine, alcohol and nicotine. But LY379268 also attenuated food self-administration at doses that attenuated the reinforcing effects of nicotine (Liechti et al., 2007). Thus, the effects of the mGlu2/3 agonist were not specific for drug rewards. Moreover, LY379268 activates both mGlu2 and mGlu3 receptors. To differentiate between the roles of these two mGlu receptors, mGlu2 selective ligands were developed. MGlu2 receptor positive allosteric modulators (PAMs) decreased self-administration of cocaine and nicotine, but not food self-administration (Jin et al., 2010; Sidique et al., 2012; Dhanya et al., 2014). Further, blockade of mGlu2 receptors using a mGlu2 antagonist (LY341495) facilitated alcohol consumption (Zhou et al., 2013). Together, these data support a role for mGlu2 receptors in drug reward. The role of mGlu3 receptors in drug reward, in contrast, needs to be further explored. Availability of selective ligands for mGlu2 and mGlu3 receptors in the future will help better understand the function of mGlu2 and mGlu3 receptors in drug reward.

Blockade of glutamate transmission via activation of mGlu7 receptors attenuated cocaine self-administration (Li et al., 2009) and alcohol-induced CPP (Bahi et al., 2012). The role of mGlu7 receptors in nicotine and heroin reward remains to be investigated. Similarly, activation of mGlu8 receptors attenuated alcohol self-administration, suggesting that these receptors are involved in the reinforcing effects of nicotine (Bäckström and Hyytia, 2005). The role of mGlu8 receptors in the rewarding effects of other drugs of abuse has not yet been explored.

Glutamate transmission can also be decreased via activation and/or upregulation of the glutamate transporter GLT-1. Administration of a GLT-1 activator decreased cocaine-induced CPP (Nakagawa et al., 2005). Further, repeated administration of ceftriaxone, attenuated alcohol consumption in the two bottle choice paradigm (Sari et al., 2011). Ceftriaxone-induced attenuation of alcohol consumption was mediated by an upregulation of GLT-1 in the NAcc and prefrontal cortex (PFC). Moreover, administration of GPI-1046 attenuated alcohol consumption in alcohol preferring P-rats, possibly due to upregulation of GLT-1 in the NAcc (Sari and Sreemantula, 2012). Alcohol consumption in P rats was also reduced after administration of 5-methyl-1-nicotinoyl-2-pyrazoline (MS-153) (Alhaddad et al., 2014b). This MS-153-induced attenuation of alcohol consumption was possibly mediated by an upregulation of GLT-1 and/ or xCT (light chain of the cystine-glutamate exchanger) in several brain sites including the NAcc, amygdala and hippocampus (Alhaddad et al., 2014b; Aal-Aaboda et al., 2015). Further, these studies also showed that MS-153 mediated upregulation was mediated by activation of p-Akt and NF-kB pathways. In summary, these findings suggest that efficient clearance of synaptic glutamate helps in reducing the rewarding effects of cocaine and alcohol.

Glutamate transmission can also be regulated by manipulating glutamate release and uptake via glial cells. Activation of the cystine-glutamate exchanger, using *N*-acetylcysteine, increases extrasynaptic glutamate levels. Surprisingly, *N*-acetylcysteine attenuated nicotine self-administration in rats (Ramirez-Niño et al., 2013). One possible explanation for the reported findings is that the increase in extrasynaptic glutamate levels induced by *N*-acetylcysteine in turn stimulates the presynaptic mGlu2/3 receptors, which then reduces synaptic glutamate release (Moussawi and Kalivas, 2010).

Another way to attenuate glutamate transmission is by blocking calcium ion channels located on presynaptic glutamate terminals. Such drugs which decrease presynaptic glutamate release may be useful in attenuating the rewarding effects of drugs of abuse. Gabapentin, a FDA-approved antiepileptic medication, reduces release of several neurotransmitters, including glutamate, by inhibiting the α2δ-1 subunit of voltage-gated calcium channels (Gee et al., 1996; Fink et al., 2000). Whole-cell patch clamp recordings showed that gabapentin attenuated electrically stimulated excitatory neurotransmission in NAcc slices obtained from cocaine-experienced animals (Spencer et al., 2014). Further, the same study showed that cocaine self-administration increased expression of the α2δ-1 subunit in the NAcc. In addition, α2δ-1 subunit expression increased in the cerebral cortex after exposure to alcohol, methamphetamine, and nicotine (Hayashida et al., 2005; Katsura et al., 2006; Kurokawa et al., 2011). A recent study reported that gabapentin attenuated methamphetamine-induced CPP (Kurokawa et al., 2011). However, the effects of gabapentin or other α2δ-1 subunit antagonists on the rewarding effects of other drugs of abuse have not been directly assessed. Another FDA-approved antiepileptic medication, lamotrigine, also decreases glutamate release from presynaptic glutamate terminals (Cunningham and Jones, 2000). In rats, lamotrigine attenuated cocaine-induced lowering of brain reward thresholds (Beguin et al., 2012). But, this effect of lamotrigine was seen at doses that elevated brain reward thresholds when administered alone, suggesting that lamotrigine may have induced an aversive state in animals. Nonetheless, in clinical trials, lamotrigine did not alter subjective effects of cocaine (Winther et al., 2000). The effects of lamotrigine on the rewarding effects of other drugs of abuse have not been systematically explored. Nonetheless, it must be remembered that in addition to inhibiting glutamate release, lamotrigine has other mechanisms of action (Yuen, 1994).

In summary, mounting evidence suggests that compounds that block glutamate transmission attenuate the rewarding effects of drugs of abuse. Both ionotropic and mGlu receptors have been implicated in mediating the rewarding effects of the different drugs of abuse. A better understanding of the role of Group III metabotropic receptors in drug reward is necessary and will likely be possible as good pharmacological ligands for these receptors become available.

### Future directions: glutamate and drug reward

Glial cells in the extrasynaptic space are key players in the regulation of glutamate transmission and neuronal communication (Scofield and Kalivas, 2014). Consequently, modulation of glial function may be able to attenuate the rewarding effects of drugs of abuse. In support of this hypothesis, administration of ibudilast, a glial cell modulator, attenuated alcohol intake in a two bottle choice paradigm in selectively bred alcohol-preferring rats, suggesting that it decreases the reinforcing effects of alcohol (Bell et al., 2015). Although the effects of ibudilast on the rewarding effects of heroin have not been evaluated, ibudilast attenuated morphine-induced CPP, and increase in NAcc dopamine after morphine administration (Hutchinson et al., 2007; Bland et al., 2009). The mechanism of action of ibudilast is not fully understood, and it is not clear how ibudilast alters glutamate transmission. It also remains to be determined if ibudilast can affect the rewarding effects of other drugs of abuse, such as cocaine and nicotine. Nevertheless, modulating the rewarding effects of drugs of abuse by influencing the function of glial cells may be a critical future strategy.

Also of interest is the fact that glutamate receptors cross-talk either directly or via signal transduction pathways with ion channels (e.g., calcium channels) and receptors for other neurotransmitters such as serotonin, dopamine, and GABA (Kubo et al., 1998; Cabello et al., 2009; Molinaro et al., 2009). Therefore, one way to reduce glutamate transmission to attenuate the rewarding effects of drugs of abuse could be via exploitation of heterooligomeric complexes formed between glutamate and non-glutamate receptors or ion channels (Duncan and Lawrence, 2012). A recent study has reported cross-talk between mGlu2 receptors and 5HT_2C_ receptors (González-Maeso et al., 2008). Indeed, blockade of 5HT_2C_ receptors in the NAcc attenuated cocaine-induced increase in glutamate levels in cocaine-experienced animals (Zayara et al., 2011). Similarly, there is evidence of interaction between mGlu5 receptors and adenosine A_2A_ receptors (Ferre et al., 2002). Administration of an adenosine A_2A_ receptor antagonist attenuated an increase in striatal glutamate levels observed after mGlu5 receptor agonist administration (Pintor et al., 2000). Taken together, these studies suggest that glutamate signaling can be manipulated via non-glutamate receptors. However, much work still remains to understand the interaction of glutamate receptors with non-glutamate receptors, and it is not known if these receptor complexes can be manipulated to attenuate the rewarding effects of drugs of abuse.

Drugs of abuse like alcohol and cocaine increase expression of certain microRNAs (miRNAs) in brain regions associated with reward (Hollander et al., 2010; Li et al., 2013; Tapocik et al., 2014). In fact, manipulating expression of miRNAs can attenuate the rewarding effects of cocaine and alcohol (Schaefer et al., 2010; Bahi and Dreyer, 2013). MiRNAs also regulate glutamate receptor expression and function (Karr et al., 2009; Kocerha et al., 2009). In addition, some miRNAs, such as miRNAs-132 and 212, are specifically regulated by mGlu receptors, but not ionotropic receptors (Wibrand et al., 2010). Therefore, future studies may need to explore if the rewarding effects of drugs of abuse can be attenuated by manipulating miRNAs that regulate glutamatergic signaling. Nevertheless, one must be cautious, as manipulating miRNA expression may affect the functioning of multiple targets and may not be restricted to glutamate signaling (Bali and Kenny, 2013).

Drug addiction in humans is frequently initiated by the consumption of drugs during adolescence. In fact, in humans, the processing of rewards differs between adults and adolescents (Fareri et al., 2008). Similarly, several studies have reported differences in the rewarding effects of drugs of abuse between adult and adolescent rats (Philpot et al., 2003; Badanich et al., 2006; Zakharova et al., 2009; Doherty and Frantz, 2012; Schramm-Sapyta et al., 2014; Lenoir et al., 2015). Additionally, gender influences drug addiction in humans (Rahmanian et al., 2011; Bobzean et al., 2014; Graziani et al., 2014) and the rewarding effects of drugs of abuse in animals (Lynch and Carroll, 1999; Russo et al., 2003a,b; Torres et al., 2009; Zakharova et al., 2009). Further, alcohol differentially affects basal glutamate levels in male compared to female rats (Lallemand et al., 2006, 2011). However, the impact of age and gender, either alone or combined, on the role of glutamate in drug reward has not been systematically explored. Future studies addressing the impact of age and gender on glutamate transmission and drug reward will enhance our understanding of the role of glutamate in drug addiction.

## Drugs of abuse and glutamate transmission in specific brain regions associated with drug reward

The rewarding effects of drugs of abuse are mediated by mesolimbic dopaminergic neurons, which originate in the VTA and project to several limbic and cortical sites such as the NAcc, amygdala and prefrontal cortex (PFC). Amongst these regions, the NAcc is a major terminal region of dopaminergic neurons originating in the VTA. Systemic administration of cocaine, nicotine, alcohol, and heroin increase dopamine levels in the NAcc (Di Chiara and Imperato, 1988; Wise et al., 1995a,b; Doyon et al., 2003; Kosowski et al., 2004; D'Souza and Duvauchelle, 2006; D'souza and Duvauchelle, 2008; Howard et al., 2008; D'Souza et al., 2011). This drug-induced increase in activity of the mesocorticolimbic dopaminergic neurons is hypothesized to mediate the rewarding effects of all drugs of abuse, including nicotine, cocaine, alcohol, and heroin (Wise, 1987; Koob, 1992b; Koob and Volkow, 2010; Salamone and Correa, 2012). Interestingly, blockade of glutamatergic transmission via systemic administration of glutamate receptor ligands attenuated cocaine- and nicotine-induced increases in NAcc dopamine (see Table 2). Both the VTA and NAcc receive extensive glutamatergic afferents. The next section will therefore describe the effects of drugs of abuse on glutamatergic transmission in the VTA and NAcc. Further, we will discuss effects of pharmacological manipulation of glutamate transmission in the VTA and NAcc on drug reward. While glutamate transmission in other brain regions may also be associated with reward, in this review we will restrict our discussion to the VTA and NAcc.

**Table 2:** **Effects of pharmacological manipulation of glutamate transmission on drug-induced increase of nucleus accumbens dopamine levels using *in vivo* microdialysis**.

**Drug of abuse**	**Glutamate receptor/Transporter**	**Pharmacological class**	**Compound**	**Status of animal**	**Dose and mode of drug administration**	**Species/Strains**	**Effect on dopamine levels**	**References**
Cocaine	NMDA	Antagonist	MK-801	Cocaine-naive	15 mg/kg; i.p.	Rats/Sprague-Dawley	No effect	Wolf et al., 1994
		Antagonist	Dizocilpine	Cocaine-naive	15 mg/kg; i.p.	Rats/Sprague-Dawley	No effect	Pierce et al., 1997
	mGlu2/3	NAAG peptidase inhibitor	2-PMPA	Cocaine-experienced	10 mg/kg; i.p.	Rats/Long-Evans	Decreased	Xi et al., 2010
	mGlu7	Positive allosteric modulator	AMN082	Cocaine-experienced	10 mg/kg; i.p.	Rats/Long-Evans	No effect	Li et al., 2009
Nicotine	NMDA	Antagonist	CGP39551	Nicotine-naive	0.6 mg/kg; s.c.	Rats/Wistar	Decreased	Kosowski et al., 2004
	NMDA (NR2B selective)	Antagonist	Ro 25-6981	Nicotine-naive	0.1 mg/kg; s.c.	Rats/Wistar	Increased	Kosowski and Liljequist, 2004
	AMPA	Antagonist	ZK200775	Nicotine-naive	Nicotine-naive	Rats/Wistar	Decreased	Kosowski et al., 2004
		Antagonist	NBQX	Nicotine-naive	Nicotine-naive	Rats/Wistar	No effect	Kosowski et al., 2004
	mGlu2/3	Agonist	LY379268	Nicotine-experienced	0.03 mg/kg; single intravenous self-infusion	Rats/Wistar	Decrease	D'Souza et al., 2011
	mGlu2/3	Agonist	LY379268	Nicotine-naive	0.4 mg/kg s.c.	Rats/Wistar	No effect	D'Souza et al., 2011
	mGlu5	Negative allosteric modulator	MPEP	Nicotine-experienced	0.4 mg/kg s.c.	Rats/Sprague-Dawley	Decreased	Tronci and Balfour, 2011

### VTA

The VTA receives extensive glutamatergic inputs from different limbic, cortical, and subcortical nuclei, such as the amygdala, PFC, lateral habenula, lateral hypothalamus, ventral pallidum, medial septum, septofimbrial nucleus, and ventrolateral bed nucleus of the stria terminalis (Geisler and Zahm, 2005; Geisler and Wise, 2008; Watabe-Uchida et al., 2012). VTA dopaminergic neurons also receive glutamatergic projections from brainstem structures, such as the mesopontine reticular formation, laterodorsal tegmental, and pedunculopontine tegmental nucleus, cuneiform nucleus, median raphe, and superior colliculus (Geisler and Trimble, 2008). These glutamatergic inputs regulate the burst firing of VTA dopaminergic neurons and thus can regulate drug-induced rewarding effects (Taber et al., 1995; Overton and Clark, 1997). Moreover, direct injection of glutamate receptor antagonists into the VTA attenuated nicotine-induced increase in NAcc dopamine (Schilstrom et al., 1998; Fu et al., 2000).

#### Drugs of abuse and VTA glutamate levels

Effects of drugs of abuse on VTA glutamate levels is shown in Table 3. Cocaine administration increased VTA glutamate levels in both cocaine-naïve and -experienced animals. In cocaine-experienced animals, cocaine-induced increase in VTA glutamate levels was observed at doses that are associated with the rewarding effects of cocaine (Kalivas and Duffy, 1998; Zhang et al., 2001). Conversely, in cocaine-naïve animals, the increase in glutamate was brief and less pronounced compared to that seen in cocaine-experienced animals (Kalivas and Duffy, 1995; Zhang et al., 2001). The facilitation of glutamate release following repeated cocaine exposure is mediated by an upregulation of D1 receptor signaling and was attenuated by blockade of D1 dopamine receptors (Kalivas and Duffy, 1998; Kalivas, 2009). Consistent with the above studies, increase in VTA glutamate levels was observed after cocaine self-administration in cocaine-experienced animals, but not in cocaine-naïve animals with saline self-administration experience (You et al., 2007). However, the increase in VTA glutamate levels in cocaine-experienced animals was transient and was not seen throughout the cocaine self-administration period. Interestingly, the increase in VTA glutamate levels in cocaine-experienced animals was also observed after self-administration of saline, suggesting that VTA glutamate release may be linked to expectation of cocaine and induced by cocaine-associated cues (Wise, 2009). Intriguingly, increase in VTA glutamate levels was also observed in cocaine-experienced animal after an intraperitoneal injection of cocaine methiodide, which does not cross the blood brain barrier (Wise et al., 2008). These data support the hypothesis that peripheral interoceptive cues associated with cocaine may be sufficient for VTA glutamate release. However, further work is required to determine if changes in VTA glutamate levels observed after administration of cocaine and/or cocaine-associated cues results from activation of similar or different brain inputs to the VTA.

**Table 3:** **Effects of drugs of abuse on glutamate levels in specific brain regions**.

**Drug of abuse**	**Brain region**	**Neurochemical technique**	**Status of animal**	**Species/Strain**	**Dose and mode of administration**	**Effect on glutamate levels**	**References**
Cocaine	NAcc	*In vivo* microdialysis	Drug-naive	Rats/Sprague-Dawley	30 mg/kg, i.p.	Increased	Reid et al., 1997
				Rats/Sprague-Dawley	30 mg/kg, i.p.	Increased	Smith et al., 1995
				Rats/Sprague-Dawley	10 mg/kg, i.p.	No effect	Zhang et al., 2001
				Rats/Lewis	1–4 mg/kg, i.v. self-administration; single dose	No effect	Miguéns et al., 2008
				Rats/Long Evans	1 mg/kg, i.v.; multiple yoked administration	No effect	Suto et al., 2010
			Cocaine-experienced	Rats/Sprague-Dawley	15 mg/kg, i.p.	Increased	Pierce et al., 1996a
				Rats/Sprague-Dawley	15 mg/kg, i.p.	Increased	Reid and Berger, 1996
				Rats/Sprague-Dawley	10 mg/kg, i.p.	Increased	Zhang et al., 2001
				Rats/Lewis	1 mg/kg, i.v. self-administration; multiple doses	Increased	Miguéns et al., 2008
				Rats/Long Evans	10 mg/kg, i.p.	Increased	Li et al., 2010
				Rats/Long Evans	1 mg/kg, i.v.; multiple self-administration	Increased	Suto et al., 2010
				Rats/Long Evans	1 mg/kg, i.v.; multiple yoked administration	Decreased	Suto et al., 2010
		Voltammetry	Drug-naive	Rats/Long Evans	1 mg/kg, i.v. self-administration; single dose	Increased	Wakabayashi and Kiyatkin, 2012
	VTA	*In vivo* microdialysis	Drug-naive	Rats/Sprague-Dawley	15 mg/kg, i.p.	Increased	Kalivas and Duffy, 1995
				Rats/Sprague-Dawley	10 mg/kg, i.p.	Increased	Zhang et al., 2001
				Rats/Long Evans	1 mg/kg, i.v. self-administration; multiple doses	No effect	You et al., 2007
			Cocaine-experienced	Rats/Sprague-Dawley	15 mg/kg, i.p.	Increased	Kalivas and Duffy, 1998
				Rats/Sprague-Dawley	10 mg/kg, i.p.	Increased	Zhang et al., 2001
				Rats/Long Evans	1 mg/kg, i.v. self-administration; multiple doses	Increased	You et al., 2007
Nicotine	NAcc	*In vivo* microdialysis	Drug-naive	Rats/Sprague-Dawley	0.3–0.6 mg/kg, i.p.	Increased	Reid et al., 2000
				Rats/Wistar	0.3 mg/kg	Increased	Lallemand et al., 2006
				Rats/Sprague-Dawley	0.5 mg/kg, i.p.	Increased	Liu et al., 2006
				Rats/Wistar	0.15–0.6 mg/kg, i.p.	Increased	Kashkin and De Witte, 2005
				Rats/Sprague-Dawley	0.09–0.135 mg/kg i.v.	Increased	Fu et al., 2000
			Nicotine-experienced	Rats/Wistar	0.3 mg/kg oral gavage	Increased	Lallemand et al., 2006
		*In vivo* voltammetry	Drug-naive	Rats/Long Evans	0.03 mg/kg i.v.	Increased	Lenoir and Kiyatkin, 2013
	VTA	*In vivo* microdialysis	Drug-naive	Rats/Sprague-Dawley	0.09–0.135 mg/kg i.v.	Increased	Fu et al., 2000
		*In vivo* voltammetry	Drug-naive	Rats/Long Evans	0.03 mg/kg i.v.	Increased	Lenoir and Kiyatkin, 2013
Alcohol	NAcc	*In vivo* microdialysis	Drug-naive	Rats/Lewis	0.5 g/kg; i.p.	No effect	Selim and Bradberry, 1996
					1 g/kg; i.p.	Increased	Selim and Bradberry, 1996
					2 g/kg; i.p.	No effect	Selim and Bradberry, 1996
				Rats/Wistar	1-3 g/kg; i.p.	No effect	Dahchour et al., 1994
				Rats/Sprague-Dawley	0.5 g/kg; i.p.	Increased	Moghaddam and Bolinao, 1994
					1 g/kg; i.p.	Increased	Moghaddam and Bolinao, 1994
					2 g/kg; i.p.	Decreased	Moghaddam and Bolinao, 1994
				Rats/Sprague-Dawley	2 g/kg; i.p.	Decreased	Yan et al., 1998
				High alcohol sensitive rats; Colorado Health Science center	1 g/kg; i.p.	Increased	Quertemont et al., 2002
				Low alcohol sensitive rats; Colorado health science center	2 g/kg; i.p.	Increased	Dahchour et al., 2000
			Alcohol-experienced	Rats/Wistar	3 g/kg; oral gavage	Increased	Lallemand et al., 2011
				Mice/C57BL/6J m	2 g/kg; i.p.	Increased	Kapasova and Szumlinski, 2008
				Mice/DBA2/J	2 g/kg; i.p.	No effect	Kapasova and Szumlinski, 2008
	VTA	*In vivo* microdialysis	Drug-naive	Alcohol preferring rats/ National Institute for Health and Welfare, Helsinki, Finland	1-2 g/kg; i.p.	No effect	Kemppainen et al., 2010
			Drug-naive	Rats/Wistar (female)	0.5 mg/kg; i.p.	Increased	Ding et al., 2012
				Rats/Wistar (female)	1 mg/kg; i.p.	No effect	Ding et al., 2012
				Rats/Wistar (female)	2 g/kg; i.p.	Decreased	Ding et al., 2012
			Alcohol-experienced	Rats/Wistar (female)	2 g/kg; i.p.	Decreased	Ding et al., 2012
Heroin	NAcc	*In vivo* microdialysis	Drug-naive	Rats/Sprague-Dawley	0.25 mg/kg; s.c.	No effect (small non-significant decrease)	Lalumiere and Kalivas, 2008
	VTA	*In vivo* microdialysis	Heroin-experienced	Long-Evans	300 ng/h; self-infusion (cumulative)	No effect	Wang et al., 2012
	Ventral pallidum	*In vivo* microdialysis	Heroin-experienced	Rats/Wistar	0.02 mg/kg, intravenous self-infusion; 15 infusions (cumulative)	Increase	Caille and Parsons, 2004

In accordance with the effects of cocaine on VTA glutamate levels, an increase in VTA glutamate levels was also observed after nicotine administration using *in vivo* microdialysis (Fu et al., 2000). Then again, Fu and colleagues observed the increase in VTA glutamate levels at doses higher than those required to observe the rewarding effects of nicotine. More recently, a study reported a transient increase in VTA glutamate levels following passive intravenous nicotine infusion (0.03 mg/kg) using *in vivo* voltammetry (Lenoir and Kiyatkin, 2013). In contrast to cocaine and nicotine, administration of alcohol did not result in an increase in VTA glutamate levels in drug-naïve alcohol-preferring rats (Kemppainen et al., 2010). Anatomically, the VTA can be divided into anterior and posterior VTA (Sanchez-Catalan et al., 2014). A more recent study has reported biphasic glutamate response in the posterior VTA to different doses of alcohol in female Wistar rats (Ding et al., 2012). Low dose (0.5 g/kg; i.p.) of alcohol resulted in a significant increase in glutamate levels compared to baseline in alcohol-naïve animals. On the other hand, high dose (2 g/kg; i.p.) of alcohol resulted in delayed decrease in VTA glutamate levels. Importantly, administration of a challenge dose of 2 g/kg (i.p.) of alcohol in alcohol-experienced animals also resulted in a decrease in VTA glutamate levels. The differences in findings between the Kemppainen et al. (2010) and Ding et al. (2012) studies is possibly due to methodological differences such as localization of probes in the VTA and strain of rats (alcohol preferring vs. Wistar rats) used in the two studies.

In contrast to cocaine, self-administration of heroin did not alter VTA glutamate levels in heroin-experienced animals (Wang et al., 2012). However, the same study also reported that self-administration of saline in heroin-experienced animals resulted in an increase in VTA glutamate levels. Taken together, these findings suggest that VTA glutamate release is responsive to heroin-associated cues but inhibited by heroin itself. It must be mentioned here that the effects of self-administered heroin on VTA glutamate levels in heroin-experienced animals was done after a single extinction session, which may have altered expectations of heroin reward. In summary, cocaine, nicotine, and alcohol administration in increase VTA glutamate levels. Next, the effects of blocking VTA glutamate transmission on the rewarding effects of drugs of abuse will be discussed.

#### VTA glutamatergic transmission and behavioral measures of drug reward

Blockade of glutamatergic transmission in the VTA via inhibition of ionotropic glutamate receptors decreased the rewarding effects of drugs of abuse (see Table 4). For example, blockade of NMDA or AMPA or both receptors in the VTA attenuated nicotine (Kenny et al., 2009) and alcohol self-administration (Rassnick et al., 1992; Czachowski et al., 2012). Furthermore, combined blockade of both NMDA and AMPA receptors in the VTA attenuated cocaine-induced CPP (Harris and Aston-Jones, 2003). Interestingly, blockade of AMPA receptors in the VTA increased heroin self-administration compared to control (Xi and Stein, 2002; Shabat-Simon et al., 2008). The increase in heroin self-administration was observed for a higher heroin dose (0.1 mg/kg/inf) that normally resulted in fewer self-administration responses. Based on this pattern of responding, the observed increase in heroin self-administration is actually hypothesized to be due to a decrease in the reinforcing effects of heroin. Interestingly, Shabat-Simon et al. (2008) showed that AMPA receptors in the anterior VTA, but not the posterior VTA, mediated the observed effects on heroin self-administration. Overall, the role of AMPA receptors in the VTA on the reinforcing effects of heroin are not clear, and further studies using a progressive ratio schedule, which measures the motivation of the animal to work for an infusion of heroin, are needed. In summary, glutamate transmission via ionotropic receptors in the VTA mediates the rewarding effects of alcohol, cocaine, nicotine, and possibly heroin.

**Table 4:** **Effects of pharmacological manipulation of glutamatergic transmission after intracranial administration in specific brain sites on drug reward**.

**Drug of abuse**	**Brain region**	**Glutamate receptor/Transporter**	**Pharmacological class**	**Compound**	**Species/strain**	**Model used to assess reward**	**Effect observed**	**References**
Cocaine	NAcc	NMDA	Antagonist	AP-5	Rats/Wistar	Self-administration	Increased	Pulvirenti et al., 1992
	VTA	NMDA and AMPA	Antagonist	AP-5 and CNQX	Rats/Sprague Dawley	CPP	Decreased	Harris and Aston-Jones, 2003
Nicotine	NAcc	NMDA	Antagonist	LY235959	Rats/Wistar	Self-administration	Increased	D'Souza and Markou, 2014
		mGlu2/3	Agonist	LY379268	Rats/Wistar	Self-administration	Decreased	Liechti et al., 2007
		mGlu5	Negative allosteric modulator	MPEP	Rats/Wistar	Self-administration	Decreased	D'Souza and Markou, 2011
	VTA	NMDA	Antagonist	LY235959	Rats/Wistar	Self-administration	Decreased	Kenny et al., 2009
		mGlu2/3	Agonist	LY379268	Rats/Wistar	Self-administration	Decreased	Liechti et al., 2007
		mGlu5	Negative allosteric modulator	MPEP	Rats/Wistar	Self-administration	Decreased	D'Souza and Markou, 2011
Alcohol	NAcc	NMDA	Antagonist	AP-5	Rats/Wistar	Self-administration	Decreased	Rassnick et al., 1992
				AP-5	Mice/DBA/2J	CPP	Decreased	Gremel and Cunningham, 2009
				AP-5	Mice/DBA/2J	CPP	Decreased	Gremel and Cunningham, 2010
		mGlu1	Negative allosteric modulator	JNJ-16259685	Mice/C57BL6	Voluntary drinking	Decreased	Lum et al., 2014
		mGlu2/3	Agonist	LY379268	Rats/Alcohol preferring (P)	Self-administration	Decreased	Besheer et al., 2010
		mGlu5	Negative allosteric modulator	MPEP	Rats/Alcohol preferring (P)	Self-administration	Decreased	Besheer et al., 2010
		mGlu5	Negative allosteric modulator	MPEP	Mice/C57BL6	Voluntary drinking	Decreased	Cozzoli et al., 2012
	VTA	AMPA	Antagonist	CNQX	Rats/long-Evans	Self-administration	Decreased	Czachowski et al., 2012
Heroin	NAcc	NMDA	Antagonist	AP-5	Rats/Wistar	Self-administration	No effect	Pulvirenti et al., 1992
	VTA	NMDA	Antagonist	AP-5	Rats/Sprague-Dawley	Self-administration	Decreased	Xi and Stein, 2002
				Ketamine	Rats/Sprague-Dawley	Self-administration	Decreased	Xi and Stein, 2002
		AMPA	Antagonist	DNQX	Rats/Sprague-Dawley	Self-administration	Increased	Xi and Stein, 2002
				CNQX	Rats/Sprague-Dawley	Self-administration	Decreased	Shabat-Simon et al., 2008
				CNQX	Rats/Sprague-Dawley	CPP	Decreased	Shabat-Simon et al., 2008

Blockade of glutamatergic neurotransmission via metabotropic receptors in the VTA also attenuated the rewarding effects of drugs of abuse. For example, blockade of glutamate transmission in the VTA either via activation of mGlu2/3 receptors or blockade of mGlu5 receptors decreased nicotine self-administration (Liechti et al., 2007; D'Souza and Markou, 2011). Microinjections of the mGlu2/3 agonist or the mGlu5 negative allosteric modulator in these studies were directed toward the posterior VTA. Interestingly, the blockade of mGlu5 receptors in the VTA also attenuated food self-administration (D'Souza and Markou, 2011). Thus, the mGlu5 receptors in the VTA appear to mediate the reinforcing effects of both natural and drug rewards. Then again, it must be noted here that the role of the mGlu receptors in the reinforcing effects of cocaine, alcohol, and heroin has not been explored. Further, animals self-administer cocaine and alcohol directly into the posterior VTA, but not into the anterior VTA (Rodd et al., 2004, 2005). The role of glutamate in the anterior or posterior VTA in the reinforcing effects of cocaine and alcohol has not been determined.

#### Future directions: VTA heterogeneity, drug reward, and glutamate transmission

Research over the past decade has shown that the VTA dopaminergic neurons consist of different subtypes based on their inputs, distinct anatomical projections, and molecular, and electrophysiological features (Margolis et al., 2006, 2008; Lammel et al., 2011, 2012, 2014). Although a majority of the neurons in the VTA are dopaminergic, approximately 2–3% of the neurons are glutamatergic and do not express markers seen in dopaminergic and GABAergic neurons (Nair-Roberts et al., 2008). However, the precise role of these glutamatergic neurons originating in the VTA in drug-induced reward is not known. Additionally, some dopaminergic neurons in the VTA co-express tyrosine hydroxylase and the VGLUT2 and possibly co-release glutamate and dopamine at their respective terminal sites (Tecuapetla et al., 2010; Hnasko et al., 2012). In fact, optogenetic studies have shown that midbrain dopaminergic neurons that project to the NAcc, but not the dorsal striatum, co-release glutamate as a neurotransmitter (Stuber et al., 2010). It is not clear if drugs of abuse have any preferential effect on dopaminergic neurons that co-release both dopamine and glutamate in the NAcc and other terminal regions compared to neurons that release only dopamine. Further, it will be interesting to see if drug-induced firing patterns of dopaminergic neurons that co-release both glutamate and dopamine are different from dopaminergic neurons that release dopamine only. Interestingly, a recent study has shown that cocaine increases dopamine transmission but attenuates glutamate transmission in the NAcc (Adrover et al., 2014).

The glutamatergic inputs to VTA dopaminergic neurons are organized in a specific manner. For example, inputs from the PFC project onto VTA dopaminergic neurons that project back to the PFC and not to other brain regions like the NAcc (Carr and Sesack, 2000). Furthermore, glutamatergic projections from specific brain regions differentially influence dopaminergic neurons with different electrophysiological properties. For example, glutamatergic inputs from the lateral hypothalamus excite VTA dopaminergic neurons that display long-duration action potential waveforms, but inhibit VTA dopaminergic neurons that display short-duration waveforms (Maeda and Mogenson, 1981). Further, glutamatergic inputs from the PFC to the VTA dopaminergic neurons play a key role in mediating cocaine-induced behavioral responses (Pierce et al., 1998). However, the specific role of the different glutamatergic inputs to the VTA dopaminergic neurons in the rewarding effects of drugs of abuse needs to be further explored. Future studies using either optogenetic approaches or neuron specific genetic deletion of glutamate receptors will be required to address the issue.

### Nucleus accumbens

Like the VTA, the NAcc receives extensive glutamatergic projections from the PFC, amygdala, hippocampus, and thalamic nuclei (Brog et al., 1993). Glutamate can also be co-released with dopamine in the NAcc by VTA dopaminergic neurons expressing VGLUT (Hnasko et al., 2012). Together, these inputs provide spatial and contextual information, determine degree of attention allocated to stimuli, inhibit impulsive behavior, and regulate motivational and emotional responses to stimuli. Accordingly, the NAcc plays a critical role in the decision making process to obtain drug rewards. Anatomically, the NAcc is broadly divided into the core and shell subdivisions (Zahm and Brog, 1992), with the NAcc shell reported to mediate rewarding effects of drugs of abuse (Di Chiara, 2002).

#### Drugs of abuse and NAcc glutamate levels

Increase in NAcc glutamate levels in both drug-naïve and drug-experienced animals have been reported after administration of various drugs of abuse (see Table 2). Using *in vivo* microdialysis, increases in NAcc glutamate levels has been reported in drug-naïve animals after cocaine (Smith et al., 1995; Reid et al., 1997), nicotine (Reid et al., 2000; Kashkin and De Witte, 2005; Lallemand et al., 2006; Liu et al., 2006), and alcohol administration (Moghaddam and Bolinao, 1994; Selim and Bradberry, 1996; Dahchour et al., 2000). Then again, the increases in NAcc glutamate levels after cocaine and alcohol were seen at doses higher than those required to produce rewarding effects. In fact, at doses that produce rewarding effects, no change in glutamate levels was observed after cocaine and alcohol administration in drug-naïve animals (Dahchour et al., 1994; Selim and Bradberry, 1996; Zhang et al., 2001; Miguéns et al., 2008). Glutamate can be neurotoxic and result in cell death (Choi, 1988). Therefore, the increase in glutamate in response to high drug doses possibly suggests neurotoxic effects rather than rewarding effects. One possible reason why studies did not detect an increase in glutamate levels after administration of rewarding doses of cocaine could be due to slow temporal resolution of the *in vivo* microdialysis technique. A recent study using voltammetry, which has faster temporal resolution, was able to detect a transient increase in glutamate in the NAcc after intravenous self-administration of a rewarding dose of cocaine (Wakabayashi and Kiyatkin, 2012). In contrast to drug-naïve animals, the increase in NAcc glutamate levels in cocaine- and alcohol-experienced animals after administration of cocaine and alcohol was observed at doses frequently used to assess rewarding effects of cocaine and alcohol, respectively (Pierce et al., 1996a; Reid and Berger, 1996; Zhang et al., 2001; Kapasova and Szumlinski, 2008; Miguéns et al., 2008; Suto et al., 2010; Lallemand et al., 2011). This is possibly due to drug-induced plasticity at presynaptic glutamatergic terminals (Kalivas, 2009). Interestingly, basal NAcc glutamate levels were lower in cocaine-experienced animals compared to saline-experienced animals (Suto et al., 2010). Further, the same study showed opposite effects of cocaine self-administration vs. yoked cocaine administration on NAcc glutamate levels in rats trained to self-administer cocaine. Cocaine-self-administration increased NAcc glutamate levels in cocaine-experienced rats. In contrast, yoked administration of cocaine in the presence of cocaine-associated cues lowered NAcc glutamate levels below baseline in cocaine-experienced rats. Together, these data suggest that expectancy of cocaine reward in response to an operant behavior can influence cocaine-induced glutamate levels.

Remarkably, high doses of alcohol produced a decrease in NAcc glutamate levels (Moghaddam and Bolinao, 1994; Yan et al., 1998). This decrease could possibly be due to an increase in alcohol-mediated GABAergic inhibition of presynaptic glutamate terminals. The effects of alcohol on NAcc glutamate levels may be determined by behavioral sensitivity of animals to alcohol. For example, alcohol had opposite effects on NAcc glutamate levels in drug-naïve rats bred specifically for their high vs. low sensitivity to the behavioral effects of alcohol (Dahchour et al., 2000). Rats with low sensitivity to the behavioral effects of alcohol showed an increase in NAcc glutamate levels, while rats with high sensitivity to alcohol showed a decrease in NAcc glutamate levels (but also see Quertemont et al., 2002). In line with these findings, a differential effect of alcohol on the NAcc glutamate levels was also observed in alcohol-experienced mice with differential susceptibility to the behavioral effects of alcohol (Kapasova and Szumlinski, 2008). Thus, alcohol-induced glutamate release may be determined by genetic underpinnings that determine susceptibility to alcohol dependence.

A differential effect of alcohol on glutamate transmission based on gender has also been reported (Lallemand et al., 2011). For example, using a model intended to mimic binge drinking in teenagers, Lallemand et al. (2011) reported increased alcohol-induced glutamate levels in the NAcc in alcohol-experienced male rats but not female rats. It must be highlighted here that chronic alcohol exposure significantly elevated basal glutamate levels in female, but not male rats. Gender differences in alcohol metabolism have been reported across species, including rats (Sutker et al., 1983; Iimuro et al., 1997; Robinson et al., 2002). It is not clear if differences in alcohol metabolism between male and female rats could account for the differences in alcohol on NAcc glutamate levels and the precise mechanism for this differential effect of alcohol on basal glutamate levels needs to be determined. Similarly, differences in basal glutamate levels have been reported between male and female rats after chronic nicotine exposure (Lallemand et al., 2006, 2011). Studies are needed to determine if there are sex-dependent differences in glutamate release after chronic cocaine exposure.

In contrast to the drugs described above, administration of heroin does not increase NAcc glutamate levels in drug-naïve rats. In fact, researchers showed a slight decrease (non-significant) in NAcc glutamate levels after heroin administration (Lalumiere and Kalivas, 2008). In contrast, acute morphine injection in drug-naïve rats increased NAcc glutamate levels. However, an increase in glutamate levels was observed downstream from the NAcc in the ventral pallidum during heroin self-administration (Caille and Parsons, 2004). Overall, the effects of heroin on NAcc glutamate levels are not clear.

Interestingly, heroin-associated cues have been shown to increase glutamate levels in the NAcc core (Lalumiere and Kalivas, 2008). In addition, in cocaine-experienced animals, presentation of cues predictive of cocaine availability increased NAcc glutamate levels (Hotsenpiller et al., 2001; Suto et al., 2010, 2013). Moreover, glutamate levels in the NAcc core were depressed on presentation of cues predicting unavailability of cocaine (Suto et al., 2013). Taken together, these data suggest that NAcc glutamate levels can be modulated by cues predicting availability or unavailability of cocaine. However, it is not known if the temporal resolution (transient vs. sustained), localization (synaptic vs. extrasynaptic) of glutamate release and activity of glutamatergic afferents to the NAcc in response to drug and/or drug-associated cues is similar or different. Future studies may need to address these issues.

In summary, repeated exposure to drugs of abuse facilitates drug-induced increase in NAcc glutamate levels compared to drug-naïve animals. Nevertheless, more work is required to determine factors [e.g., genetic factors, effects of gender (male vs. female), location (synaptic vs. extrasynaptic), temporal resolution (transient vs. sustained), precise glutamatergic inputs activated] that can influence changes in NAcc glutamate levels in response to drug and/or drug-associated cues.

#### NAcc glutamatergic transmission and behavioral measures of drug reward

Blockade of glutamate neurotransmission in the NAcc had a differential effect on rewarding effects of drugs of abuse (see Table 4, discussed below). Blockade of NMDA receptors in the NAcc decreased both alcohol self-administration and alcohol-induced CPP (Rassnick et al., 1992; Gremel and Cunningham, 2009, 2010). Together, these studies suggest that NMDA-mediated glutamate transmission in the NAcc mediates the rewarding effects of alcohol.

In contrast, blockade of NMDA receptors into the NAcc using the competitive NMDA receptor antagonist LY235959 increased nicotine self-administration under a fixed-ratio schedule (D'Souza and Markou, 2014). This effect was seen specifically in the NAcc shell and not in the NAcc core. Furthermore, LY235959 injections into the NAcc shell decreased food self-administration, suggesting that the effects of LY235959 were specific for the reinforcing effects of nicotine. Moreover, LY235959 injections into the NAcc shell increased nicotine self-administration under a progressive-ratio schedule, suggesting that blockade of the NMDA receptors increased motivation to self-administer nicotine. Motivation to self-administer nicotine under a progressive-ratio schedule of reinforcement was also increased after local infusion of the α7 nAChR antagonist α-conotoxin ArIB into the NAcc shell and decreased after infusion of the α7 nAChR agonist PNU282987 into the NAcc shell (Brunzell and McIntosh, 2012). Nicotine binds to α7 nAChRs located on presynaptic glutamatergic terminals and increases glutamatergic transmission, and blockade of α7 nAChRs decreases glutamate transmission. In line with the above findings, blockade of NMDA receptors in the NAcc shell using another competitive antagonist, AP-5, resulted in increased cocaine self-administration under a fixed-ratio schedule (Pulvirenti et al., 1992). But the same study showed no effect of the same NMDA receptor antagonist in the NAcc on heroin self-administration. Taken together, decreased glutamate transmission via NMDA receptors in the NAcc shell increases the reinforcing effects of stimulants like nicotine and cocaine, but not that of depressants like alcohol and heroin.

The precise mechanism for the increase in the reinforcing effects of nicotine after injection of NMDA receptor antagonists in the NAcc is not fully understood. One potential mechanism could be that NMDA receptor antagonists inhibit medium spiny neurons that send inhibitory projections directly back to the VTA dopaminergic neurons (Kalivas, 1993). In other words, injections of NMDA antagonists in the NAcc increase the firing of VTA dopaminergic neurons. This hypothesis will need to be tested in future studies. Interestingly, rats have been shown to self-administer both competitive and non-competitive NMDA antagonists directly in the NAcc (Carlezon and Wise, 1996). In summary, blockade of NMDA-mediated glutamate transmission in the NAcc can have differential effects on drug reward depending on the drug being studied. Future studies using subunit specific NMDA receptor ligands may be needed to fully understand the role of NAcc NMDA receptors in drug reward. Studies are also required to address mechanisms responsible for the differential effect of NMDA-mediated glutamate transmission in the reinforcing effects of nicotine, cocaine, heroin, and alcohol.

It is intriguing that studies assessing the effects of blockade of AMPA receptors in the NAcc on drug reward are lacking. Therefore, it is not known if the effects of NMDA receptor blockade on drug reward can be extended to other ionotropic receptor-mediated glutamate transmission. It is very likely that the AMPA receptor blockade has different effects from that of NMDA receptor blockade, because numerous studies have shown differential drug-induced effects on NMDA and AMPA receptor expression and trafficking in the NAcc (Lu et al., 2003; Conrad et al., 2008; Kenny et al., 2009; Ortinski et al., 2013).

In contrast to the effects of NMDA receptor blockade described above, blockade of glutamatergic transmission via either activation of mGlu2/3 receptors or blockade of mGlu5 receptors in the NAcc shell attenuated nicotine and alcohol self-administration (Liechti et al., 2007; Besheer et al., 2010; D'Souza and Markou, 2011). Consequently, it appears that ionotropic and mGlu transmission in the NAcc may have a differential effect on the rewarding effects of nicotine. The effects of blocking of glutamatergic transmission via mGlu receptors in the NAcc on cocaine and heroin reward have not yet been studied. MGlu1 and mGlu5 receptors in the NAcc play an important role in alcohol reward. Direct injections of the mGlu1 negative allosteric modulator (JNJ-16259685) in the NAcc attenuated the rewarding effects of alcohol (Lum et al., 2014). In addition, the study showed that these mGlu1 mediated effects on alcohol reward involve the scaffolding protein homer and signaling molecule phospholipase C. Direct injections of the mGlu5 receptor negative allosteric modulator MPEP in the NAcc also decreased consumption of alcohol in mice (Cozzoli et al., 2012). Interestingly, chronic alcohol consumption in male alcohol-preferring P rats resulted in decreased expression of xCT in the NAcc, suggesting that manipulation of the exchanger in the NAcc may alter the rewarding effects of alcohol (Alhaddad et al., 2014a). Moreover, based on results obtained after systemic administration of drugs that modulate glutamate transmission, studies exploring the role in drug reward of the cystine-glutamate exchanger, GLT-1 transporters, mGlu8 and mGlu7 receptors in the NAcc are warranted.

#### Future directions: NAcc heterogeneity, drug reward, and glutamate transmission

The NAcc is composed of medium spiny GABAergic neurons (~90–95%) mixed with GABA and cholinergic interneurons. The medium spiny GABAergic neurons project to several brain regions, including the ventral pallidum and VTA, that are responsible for behavioral activity required to obtain rewards (Haber et al., 1990; Zahm and Brog, 1992). As described above, anatomically, the NAcc can be divided into the medial shell and lateral core (Zahm and Brog, 1992). Further, based on dopamine receptor signaling, medium spiny neurons in the striatum including the NAcc are organized into circuits expressing D1-like (includes D1 and D5 receptors) or D2-like (includes D2, D3, and D4) receptors (Gerfen, 1992). The NAcc, as described above, is a major terminal of dopaminergic neurons originating in the VTA. Glutamatergic inputs from the PFC to the NAcc terminate on dendrites of the medium spiny GABAergic neurons and form a triad with dopaminergic inputs from the VTA (Sesack and Grace, 2010). As a consequence, the activity of the different accumbal medium spiny neurons in the different accumbal subterritories is regulated by both dopamine and glutamate inputs.

*In vivo* recordings of single neuronal activity in the NAcc have shown that different sets of accumbal neurons are activated during the different phases (pre lever press, during the actual drug infusion, post lever press) of cocaine and nicotine self-administration (Peoples et al., 1999, 2004; Guillem and Peoples, 2011). Furthermore, a majority of accumbal neurons respond differently to cocaine self-administration compared to heroin self-administration (Chang et al., 1998). Moreover, different subsets of accumbal neurons are activated during consumption of natural and drug rewards (Carelli and Deadwyler, 1994; Carelli, 2002). However, the role of glutamate in the firing of accumbal neurons during drug self-administration has not been addressed. Further, the role of specific glutamate receptors in drug-induced accumbal neuronal firing has not been studied. An understanding of NMDA- and non-NMDA-mediated glutamate signaling in accumbal neuronal firing during drug self-administration may help us better interpret the evidence obtained from the different pharmacological studies described above.

## Modulation of glutamate transmission using genetic approaches and drug reward

Genetic manipulation of glutamate transmission has further strengthened our understanding of the role of both ionotropic and mGlu receptors in drug reward. For example, selective knockout of NMDA receptors located on VTA dopaminergic neurons in mice attenuated the acquisition of nicotine-induced CPP (Wang et al., 2010). Further, unlike wildtype mice, mice lacking the NR2A subunit did not acquire alcohol-induced CPP, supporting a role for NR2A subunits in alcohol reward (Boyce-Rustay and Holmes, 2006). In addition, overexpression of GluR1 in the VTA increased cocaine self-administration under a progressive-ratio schedule (Choi et al., 2011). In other words, increased AMPA receptor-mediated glutamate transmission increased motivation to self-administer cocaine. The same study also showed that expression of a mutant form of GluR1 receptors that do not increase PKA-mediated phosphorylation decreased cocaine self-administration. Overall, one can conclude that AMPA receptors contribute to both the reinforcing and motivational effects of cocaine via a PKA-mediated pathway. Interestingly, mice lacking either the GluR1 or GluR3 AMPA receptor subunits did not show a difference in alcohol consumption compared to their respective wildtype mice, suggesting that these subunits do not contribute to the reinforcing effects of alcohol (Cowen et al., 2003; Sanchis-Segura et al., 2006). Finally, mice lacking the gene for the synaptic scaffolding protein Homer 2b showed reduced alcohol preference and alcohol-induced CPP, suggesting that Homer 2b protein is involved in the reinforcing effects of alcohol (Szumlinski et al., 2005). The Homer protein is involved in interaction between NMDA and mGlu5 receptors. Thus, deleting Homer 2b proteins decreases glutamate transmission, which possibly accounts for the decreased rewarding effects of alcohol.

Mice lacking mGlu2 receptors demonstrated increased alcohol consumption, thus supporting an important role for mGlu2 receptors in alcohol reward (Zhou et al., 2013). Mice lacking mGlu5 receptors in contrast to their wildtype counterparts did not acquire cocaine self-administration, which suggests that the mGlu5 receptors play a critical role in the reinforcing effects of cocaine (Chiamulera et al., 2001). Interestingly, mice lacking mGlu5 showed decreased alcohol consumption in the two bottle choice model compared to wildtype mice (Bird et al., 2008). The same study also showed that the mGlu5 knockout mice displayed alcohol-induced CPP at a low dose (1 g/kg), which was not effective in the wildtype mice. Taken together, it appears that knockout of mGlu5 receptors increases sensitivity to alcohol. These findings are in contrast to the role of mGlu5 receptors in the reinforcing effects of alcohol, as reported by pharmacological studies using mGlu5 negative allosteric modulators described above (section Blockade of Glutamatergic Transmission and Behavioral Measures of Drug Reward). This discrepancy could be due to compensatory changes that occur following congenital manipulation of expression of a particular receptor. Knockout of mGlu4 receptors in mice did not affect alcohol consumption compared to their wildtype counterparts (Blednov et al., 2004), thus indicating that mGlu4 receptors have a limited role in the reinforcing effects of alcohol. Viral-mediated knockdown of the mGlu7 receptors in the NAcc potentiated alcohol-induced CPP and consumption of alcohol in a two bottle choice model compared to controls (Bahi, 2013). These findings suggest that lower expression of the mGlu7 receptors facilitates the reinforcing effects of alcohol. MGlu7 receptors negatively regulate glutamate transmission, and decreased expression of these receptors facilitates glutamate transmission and possibly the reinforcing effects of alcohol. Overall, findings from genetic studies involving the mGlu7 receptors are consistent with findings from pharmacological studies described above (section Blockade of Glutamatergic Transmission and Behavioral Measures of Drug Reward). In summary, findings from genetic studies confirm the role of ionotropic and mGlu receptors in drug reward. It will be interesting to see if genetic polymorphisms in glutamate receptors that make individuals more vulnerable to the rewarding effects of drugs of abuse, and subsequently to drug addiction, can be identified in humans.

## Concluding remarks

In summary, the rewarding effects of drugs of abuse play a crucial role in continued drug use and development of drug addiction. Over the years, there has been considerable progress in understanding the role of the excitatory neurotransmitter glutamate in drug reward. Drugs of abuse discussed in this review increase glutamatergic transmission in the VTA and facilitate the firing of mesocorticolimbic dopaminergic neurons. Significantly, blockade of glutamate transmission via ionotropic and mGlu receptors attenuates the rewarding effects of drugs of abuse. Further, blocking glutamate transmission in brain regions associated with reward, such as the NAcc and VTA, likewise attenuates drug reward. Finally, repeated exposure to drugs of abuse induces plasticity in several brain regions including the NAcc and VTA that leads to development of drug addiction. Taken together, these findings make glutamate transmission an alluring target for developing medications to treat drug addiction.

The ubiquitous distribution of glutamate makes targeting glutamate transmission to decrease the reinforcing effects of drug rewards very challenging. Further, it must be emphasized here that glutamate transmission is involved in many other physiological functions such as learning, memory, regulation of normal behavior and reinforcing effects of natural rewards. Hence, there is a need to develop medications that selectively attenuate the reinforcing effects of drugs of abuse without affecting other physiological functions. Nevertheless, as described in this review, the FDA has approved several medications that attenuate glutamate transmission, which suggest that glutamate transmission remains a viable target for medication development. In fact, drugs targeting the mGlu receptors are in various stages of clinical development for several CNS disorders. In conclusion, while much has been understood about the role of glutamate in drug reward, more work needs to be done to fully exploit the therapeutic potential of glutamate in drug reward and addiction.

### Conflict of interest statement

The author declares that the research was conducted in the absence of any commercial or financial relationships that could be construed as a potential conflict of interest.

## Abbreviations

ACPC: 1-aminocyclopropanecarboxylic acid
AMPA: amino-3-hydroxy-5-methyl-4-isoxazolepropionate/kainate
AP-5: (2*R*)-amino-5-phosphonovaleric acid
AMN082: *N*,*N*′-*Bis*(diphenylmethyl)-1,2-ethanediamine
BINA: Biphenyl-indanone A
CGP39551: (*E*)-(α)-2-Amino-4-methyl-5-phosphono-3-pentenoic acid ethyl ester
CPP: conditioned place preference
DNQX: 6, 7-Dinitroquinoxaline-2,3-dione
3, 4 DCPG: (*R*)-3,4-Dicarboxyphenylglycine
(+)-HA-966: -(+)-3-Amino-1-hydroxypyrrolidin-2-one
GABA: γ-aminobutyric acid
GLT: Glutamate transporter
ICSS: intracranial self-stimulation
L-701,324: 7-Chloro-4-hydroxy-3-(3-phenoxy)phenyl-2(1*H*)-quinolinone
LY37268: (1*R*,4*R*,5*S*,6*R*)-4-Amino-2-oxabicyclo[3.1.0]hexane-4,6-dicarboxylic acid
LY235959: 3*S*-[3α,4aα,6β,8aα])-decahydro-6-(phosphonomethyl)-3-isoquinolinecarboxylic acid
MK-801: (5*R*,10*S*)-(-)-5-Methyl-10, 11-dihydro-5*H*-dibenzo[*a, d*]cylcohepten-5,10-imine
mGlu: metabotropic glutamate
MPEP: 2-Methyl-6-(phenylethynyl)pyridine
MTEP: 3-((2-Methyl-1,3-thiazol-4-yl)ethynyl)pyridine
NAAG: N-acetylaspartyl glutamate
NAcc: nucleus accumbens
NMDA: *N*-methyl-D-aspartate
VTA: ventral tegmental area
xCT: light chain of the cystine-glutamate transporter
PAMs: positive allosteric modulators
2-PMPA: 2-(Phosphonomethyl)pentane-1,5-dioic acid
Ro-25-6981: (α*R*β*S*)-α-(4-Hydroxyphenyl)-β-methyl-4-(phenylmethyl)-1-piperidinepropanol
ZK200775: [[3, 4-Dihydro-7-(4-morpholinyl)-2,3-dioxo-6-(trifluoromethyl)-1(2*H*)-quinoxalinyl]methyl]phosphonic acid.
